# Growth Characterization of Single and Double *Salmonella* Methionine Auxotroph Strains for Potential Vaccine Use in Poultry

**DOI:** 10.3389/fvets.2017.00103

**Published:** 2017-06-29

**Authors:** Peter Rubinelli, Sun Ae Kim, Si Hong Park, C. Adam Baker, Steven C. Ricke

**Affiliations:** ^1^Center for Food Safety, Department of Food Science, University of Arkansas, Fayetteville, AR, United States

**Keywords:** *Salmonella* Typhimurium, vaccine, methionine auxotrophy, poultry, Δ*metR*, ΔΔ*metRmetD*

## Abstract

Poultry meat is an important source of zoonotic *Salmonella* infection. Oral vaccination of chickens with live attenuated *Salmonella* during grow-out is an attractive approach to control *Salmonella* colonization in the chicken gastrointestinal tract. In this study, we report the construction of methionine-dependent and growth of *Salmonella* Typhimurium mutant strains with methionine auxotrophy (Δ*metR* and ΔΔ*metRmetD*) and survival in chicken feed and fecal matrices. The methionine auxotroph mutant ΔΔ*metRmetD* grew slowly on L-methionine but failed to grow on D-methionine, as expected, and exhibited lower affinity for methionine compared with the isogenic parent strain (Δ*metR* single mutant) in whole-cell affinity experiments. Preliminary data conducted as part of a previously published bird challenge study indicated that the methionine auxotroph was less effective at protection in chickens to a challenge with virulent wild-type parent strain but generated greater *Salmonella*-specific serum IgG. Although the auxotroph could not sustain itself in minimal media it was able to survive when incubated in the presence of chicken and fecal material. The immune response appears promising but further work may be needed to alter low-affinity methionine transporters and methionine biosynthesis genes in combination with the knock-out of the high affinity transporter *metD* reported here to ensure timely clearance of the candidate vaccine strain.

## Introduction

As an essential amino acid for animals, methionine is an important component of animal feeds, including poultry feed (1). Methionine is one of the nutritionally limiting components of animal feeds and is limited in plant proteins. Methionine is essential for protein synthesis and serves as a source of methyl groups for the biosynthesis of lipids, biotin, nucleic acids, and polyamines (2, 3).

Methionine synthesis and uptake have been extensively investigated in *Escherichia coli* and *Salmonella* Typhimurium (4–7). Transport of methionine into the bacterial cell is mediated by both a high-affinity transporter (Km approximately 0.1 mM) and one or more low-affinity transporters (Km approximately 20–40 mM) (6). The high-affinity transporter is referred to as metD in both *E. coli* and *S*. Typhimurium, and mutants in this transporter are unable to transport D-methionine. More recently, the metD transporter gene has been sequenced and shown to consist of an operon comprised of three genes, recently named *metNIQ* (8). We have tested the hypothesis that a methionine auxotrophic strain of *S*. Typhimurium with limited uptake and synthesis of methionine can serve as an effective vaccine for the pre-harvest control of *Salmonella* in poultry. Poultry is a significant source of food-borne *Salmonella* illness in humans (9). Thus, pre-harvest control measures such as vaccination are desirable.

One approach to poultry vaccination with live attenuated *Salmonella* has focused on auxotrophy by deletion of genes encoding essential regulators of metabolism. One of these is the regulation of synthesis and uptake of methionine. The *metR* gene encodes a transcription factor of the LysR family that regulates several genes of the methionine biosynthesis pathway. The *metR* controls primarily genes involved with the last steps of methionine biosynthesis: *metF, metE*, and *metH*. The *metF* gene product produces a methyl donor, 5-methyltetrahydrofolate, which provides the terminal methyl group for methionine. Both *metE* and *metH* encode cobalamin-independent and cobalamin-dependent enzymes, respectively, that add the terminal methyl group to homocysteine to form methionine (6, 10). The *metD* deletion eliminates the high-affinity methionine transporter (7). We hypothesized that use of this mutant in combination with the *metR* deletion might further reduce the ability of a *Salmonella* vaccine strain to survive in the host by limiting methionine uptake to that of the remaining (low-affinity) methionine transporters.

In this study, single (Δ*metR*) and double (ΔΔ*metRmetD*) *S*. Typhimurium UK-1 mutants were constructed and characterized as potential vaccine strains for control of *Salmonella* colonization. Here we present preliminary data on the ΔΔ*metRmetD* unpublished part of the bird challenge study (11) and compare it with the previously published responses to the wild-type parent strain of *S*. Typhimurium, UK-1 (positive control) and a *P_BAD_*-*mviN* vaccine strain from our past research (11). The *P_BAD_*-*mviN* strain is a genetically attenuated strain that has the native promoter of the *mviN* gene (a gene required for cell wall synthesis) (12) removed and replaced with an arabinose-inducible promoter (*P_BAD_*) and the gene encoding the upstream activator, *araC*. By growing this strain in arabinose, but then washing away this medium and inoculating the washed cells orally to the chicken, the bacterium undergoes delayed lysis as cell wall synthesis shuts down (11). To assess the environmental characteristics of the methionine auxotrophs, growth kinetics in minimal medium with L-methionine as well as growth curves in D-methionine, and survival of the auxotroph strains in chicken feed and feces are presented in the current study.

## Materials and Methods

### Bacterial Strains

A wild-type *S*. Typhimurium UK-1 strain was utilized to construct potential vaccine strains. A nalidixic acid (NA) resistant *S*. Typhimurium UK-1 derived from the wild type was used as the challenge strain. The *P_BAD_*-*mviN* vaccine strain discussed in the current study for comparative purposes was generated from UK-1 as described in our previous report (11).

### Construction of ΔΔ*metRmetD S*. Typhimurium UK-1

Single and double deletion mutants affecting methionine metabolism were produced in *S*. Typhimurium strain UK-1. This strain and the plasmids for the Red recombinase system were obtained from Dr. Young Min Kwon, Department of Poultry Science, University of Arkansas (Fayetteville, AR, USA). The Red recombinase system was used for the targeted gene deletions as previously described (13). Briefly, disruption of the targeted genes was accomplished by first transforming strain UK-1 with plasmid pKD46. This plasmid confers ampicillin resistance and harbors the genes for phage lambda Red recombinase, which mediates the exchange of DNA between the gene of *Salmonella* to be deleted and the gene disruption construct. This plasmid also contains a temperature-sensitive origin of replication, which facilitates its removal following recombination. Gene disruption constructs were synthesized by amplifying a region of plasmid pKD4 (13) by polymerase chain reaction (PCR), from the P1 site (nucleotides 31–50 of pKD4) to the P2 site (nt. 1488–1507). This region consists of a central kanamycin (Kan) resistance gene (encoding aminoglycoside 3′-phosphotransferase), flanked by two FLP recognition target (FRT) sites. Genomic DNA sequences corresponding to upstream and downstream regions surrounding the appropriate target gene of strain UK-1 were subsequently introduced on either side of the FRT-Kan^R^-FRT region by overlap extension PCR (14), using the primers indicated in Table 1.

**Table 1:** Polymerase chain reaction primers used in the present study.

Primer	Sequence (5'–3')
metR-F	TCTAAATAGTTCGGCTTGCAG
metR-R	GTATAAACGTCTGATGGAGACC
metR-Up-F	AGGTACTGTATATTCCTCAAGCG
metR-Up-R	CAGCTCCAGCCTACACGATGAGACAGAGCGGATTG
metR-Dn-F	GAGGATATTCATATGGCGATCATCTGCCGTTTGTG
metR-Dn-R	GAACTATGGCGCTACCCAG
metNIQ-F1	CGACTAAGTCTTCAGCATTGG
metNIQ-F2	GATCTGCTTAGCATGGAACAAC
metNIQ-Up-R	CAGCTCCAGCCTACACGTGTACGAAGCCGCAAATAAAG
metNIQ-Dn-F	GAGGATATTCATATGGCCCCTGCTGGAACACTTTG
metNIQ-R1	TCATGTACGTAGCCGTGATCC
metNIQ-R2	CCACCTTTTATAGCTCCTGAGTAAAG

**Figure 1:**
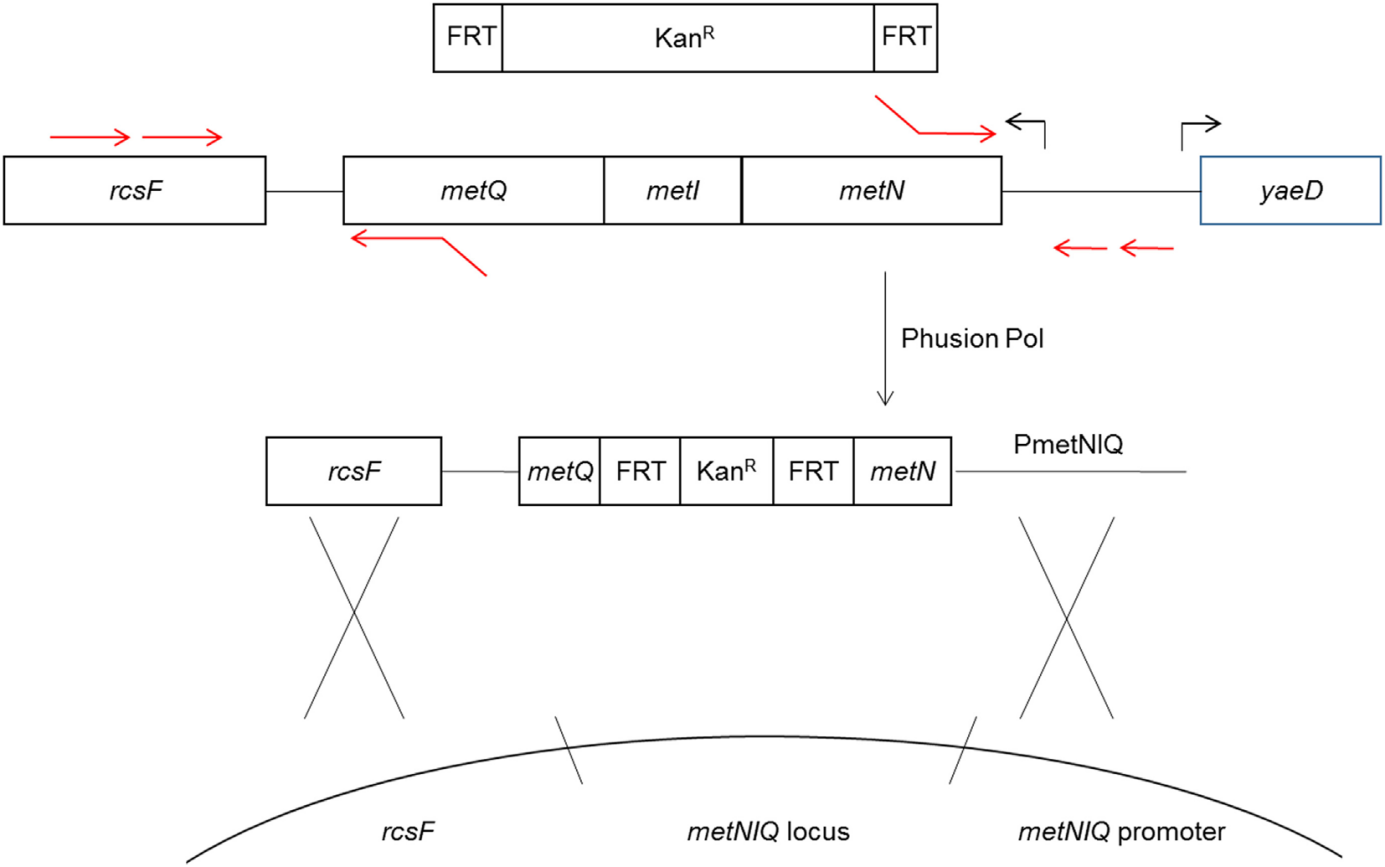
Diagram of *metD* mutant construction. The *metR* mutant was constructed in a similar fashion. The *metD* transporter consists of three subunits, encoded by the *metNIQ* operon. Primer sequences fusing the *metN* and *metQ* sequences to FRT-Kan expression cassette sequences (13) were used to delete the entire metI gene and part of the *metN* and *metQ* genes, replacing these with the FRT-Kan cassette. The resulting construct was introduced into the *metD* locus by electroporation and homologous recombination using the Red recombinase system (13).

The method for deletion of the metD transporter sequence, which is comprised of the met*NIQ* operon is shown in Figure 1. The resulting PCR products were gel purified, treated with restriction endonuclease Dpn1 to degrade any trace amount of template pKD4, gel purified again, and then electroporated into electroporation-competent UK-1:pKD4 cells that had been grown at 30°C in Luria-Bertani (LB) broth with 1 mM arabinose to induce the Red recombinase. After a 1 h incubation of the electroporated cells at 37°C in SOC medium, the electroporated cells were spread on LB/Kan agar plates and grown at 37°C overnight. Four tranformants were then streaked for isolation on LB/Kan, and tested on LB/ampicillin plates to confirm curing of plasmid pKD46.

The Kan resistance marker was subsequently removed from the genomic insertion sites, leaving a gene deletion, by introducing a second plasmid, pCP20, which expresses a second recombinase, the *S. cerevisiae* FLP recombinase and confers ampicillin resistance (15). The FLP mediates the removal of the antibiotic resistance marker by recombining the flanking FRT sites. The FLP was induced and pCP20 removed by shifting the temperature from 30 to 42°C as described previously (13). Removal of the Kan^R^ genomic insertion and pCP20 were confirmed by failure to grow on LB/Kan and LB/ampicillin, respectively, with appropriate growth on LB in parallel.

### Whole-Cell Affinity Measurements

Whole-cell affinity measurements were conducted as described previously (16). Briefly, cultures of the Δ*metR* single mutant and ΔΔ*metRmetD* double mutant were grown in M9 minimal medium + 10 μM L-methionine at 37°C for 16 h and then diluted to an OD_600_ of 0.05 in M9 minimal medium + L-methionine at 3, 7, 10, 13, 15, and 17 μM in culture tubes containing 4 ml each of minimal medium at the L-methionine concentrations indicated, and three technical replicate culture tubes were prepared at each methionine concentration. Cultures were grown at 37°C in a shaking water bath at 220 RPM, and the OD_600_ was measured every 15 min for a total of 6 h. The replicate data were then averaged and transformed for Lineweaver–Burk plots using Microsoft Excel software.

### Vaccination

Details of the vaccination challenge trial have been described elsewhere (11). Briefly male Cobb 500 broiler chicks (Siloam Springs, AR, USA) were obtained on day of hatch and randomly assigned to four pre-sterilized Horsfall units. A University of Arkansas Institutional Animal Care and Use Committee-approved protocol was used to ensure humane treatment of the chickens. Chicks vaccinated with *ΔmetRΔmetD* double mutant *Salmonella* was one treatment group (designated Group 2) of the four treatment groups (Group 1: unvaccinated, challenged; Group 3: vaccinated with the *P_BAD_*-*mviN* vaccine strain *Salmonella*, challenged), and Group 4: vaccinated with the wild-type parent strain UK-1, challenged. The vaccine and control inocula were grown for 16 h in LB broth at 37^o^C, followed by three washes in phosphate-buffered saline (PBS) and adjustment of the cell density by dilution in PBS to 5 × 10^8^ cells/ml. Chicks were orally inoculated with 1 × 10^8^ CFU *Salmonella* cells via sterile gavage needle on Day 2 post-hatch and again on Day 7 post-hatch. The unvaccinated chicks received an equal volume (0.2 ml) of sterile PBS via sterile gavage needle on Days 2 and 7 post-hatch. The challenge strain, which had been passaged repeatedly through chicks to increase its virulence followed by cryopreservation, was grown for 16 h in LB + 20 μg/ml NA. The challenge strain was then passaged twice for 8 h each passage to ensure a log phase culture. The resulting cells were then diluted to 5 × 10^8^ cells/ml with PBS and orally inoculated via sterile gavage needle to chickens at 2 weeks post-hatch with 1 × 10^8^ cells (0.2 ml).

At the time of chicken necropsy reported previously (11), ceca and ilea organs were collected aseptically and transferred to sterile sample bags, subsequently removed and transferred to 10 ml tetrathionate (TT) broth for enrichment. The TT broth was incubated for 24 h at 37°C, followed by streaking of a 10 μl loopful of the TT broth for isolation on Brilliant Green (BG) agar supplemented with 20 μg/ml NA and another 10 μl loopful on BG agar supplemented with 50 μg/ml Kan + 1 mM arabinose. Agar plates were incubated for 24 h at 37°C, examined for *Salmonella* colony appearance to enumerate the NA—resistant *S*. Typhimurium challenge strain per gram cecal content, and live vaccine strains (Kan—resistant *Salmonella* per gram of cecal content). A 0.1 g portion of cecal content was aseptically added to a sterile microtube, weighed, and then combined with nine volumes of sterile PBS to obtain a 1:10 dilution, followed by vortexing. After serial dilution in sterile PBS, mixed cecal contents were spread-plated aseptically onto selective media (BG + 20 μg/ml NA) for enumeration of the respective treatment groups. Since *S*. Typhimurium is naturally resistant to novobiocin (NO), the wild-type control NA-sensitive strain was distinguished from the NA-resistant challenge strain by direct plating of cecal contents from the wild-type-inoculated chickens on both BG + NA and BG + NO plates, and subtracting the number of colonies on BG + NA (challenge strain) from the total colonies on BG + NO (challenge strain + wild-type control strain).

### Enzyme-Linked Immunosorbent Assay (ELISA)

As described previously (11), indirect ELISA reactions were performed by placing 1 μg of *Salmonella* protein from sonicated UK-1 cells in each well of 96-well microlon medium binding microtiter plates (Grainer, Frickenhausen, Germany) after diluting to 100 μl in carbonate buffer, pH 9.6. After allowing proteins to bind for 2 h 37°C, the plates were allowed to air-dry overnight at 23°C then blocked with Superblock (Thermo Scientific, Rockford, IL, USA) 2 h, 37°C. Chicken sera from unvaccinated and vaccinated chickens from all treatment groups were serially diluted in Superblock and the plates incubated at 37°C, 2 h followed by washing four times with ELISA plate wash buffer (50 mM Tris pH 8, 140 mM NaCl, 0.05% Tween-20). Anti-chicken IgG-HRP conjugate was diluted 1:20,000 with Superblock and plates incubated 37°C, 1 h, followed by washing four times in wash buffer. The TMB substrate (KPL, Gaithersburg, MD, USA) was incubated 10 min in each well, stopped with 1 N HCl and absorbance measured in a Tecan Infinite M200 plate reader at 450 nm.

### Growth and Survival of Mutants in M9, Feed, and Fecal Broth

Wild-type parent strain and mutants Δ*metR* and Δ*metR*Δ*metD* were grown in M9 minimal medium supplemented with 10 μM L- or D-methionine as indicated for 16 h and subsequently washed three times in PBS. Five grams of chicken feed were blended with 100 ml dionized water at high speed for 3 min. The same was done for 5 g of chicken feces, separately. These blends were then filtered through cheese cloth and autoclaved. These were subsequently aliquoted into sterile culture tubes and the washed *Salmonella* was added to a final density of 1 × 10^6^ cells/ml. The cultures were incubated at 37°C, 200 RPM and growth of the cultures was monitored by spread-plating and colony counting of appropriate dilutions on LB medium at 0, 2, 5, 24, 48, 96, 264, and 504 h.

### Statistical Analysis

The enumeration of the challenge (NA-resistant marker) strain in ceca of unvaccinated and vaccinated chickens was compared by a two-tailed Student’s *t*-test, using the Microsoft Excel program.

## Results and Discussion

**Figure 2:**
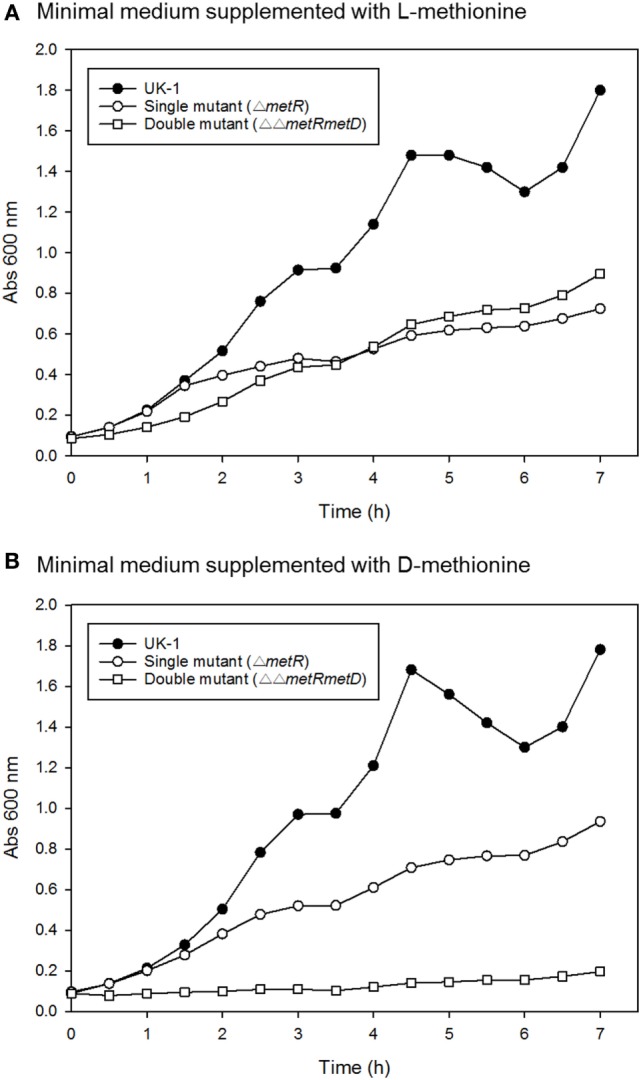
Growth curves of *S*. Typhimurium wild-type (UK-1), single (Δ*metR*), and double mutant (ΔΔ*metRmetD*) mutant in minimal medium supplemented with **(A)** L-methionine or **(B)** D-methionine.

**Figure 3:**
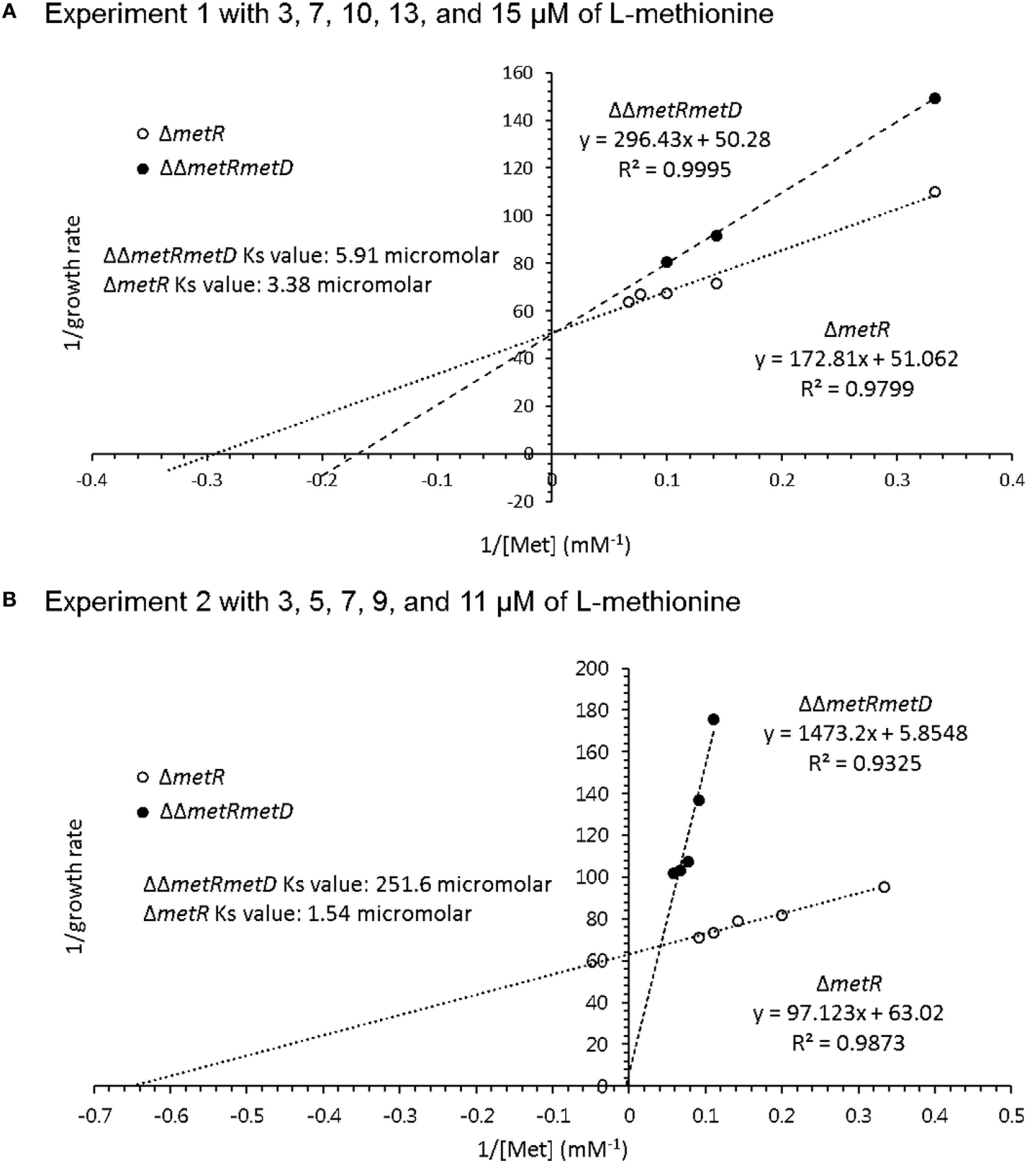
Whole-cell affinity comparing growth of Δ*metR* and ΔΔ*metRmetD* mutant in minimal medium with added L-methionine. **(A)** Experiment 1 with 3, 7, 10, 13, and 15 μM of L-methionine; **(B)** Experiment 2 with 3, 5, 7, 9, and 11 μM of L-methionine.

Growth curves of single Δ*metR* and ΔΔ*metRmetD* in M9 minimal medium supplemented with L- or D-methionine are shown in Figure 2. Both mutants exhibited reduced growth when compared with the control strain (*S*. Typhimurium UK-1) in both media with either L- or D-methionine. The double mutant exhibited little or no growth in D-methionine, as expected. The growth rates and doubling times of the mutants in M9 minimal medium are shown in Table 2. The growth rates were 10–20-fold lower than that reported by Froelich et al. for an *E. coli* methionine auxotroph (16). The whole-cell affinities of the single and double mutants for methionine are shown in Figure 3. Whole-cell affinity measurements indicated that the ΔΔ*metRmetD* double mutant had a consistently lower affinity (Ks) for L-methionine compared with the Δ*metR* single mutant (Figure 3). The Ks of the *metRmetD* double mutant was 5.90 and 251.6 μM in Experiments 1 and 2, respectively, while the corresponding values for the *metR* single mutant were 3.38 and 1.54 μM. Except for the outlier value of 251.6 μM, these values are similar to the Ks values of 6.41 and 7.00 reported by Froelich et al. for an *E. coli* methionine auxotroph, ATCC 23798 (16). The discrepancy between the much lower growth rate of the *Salmonella* auxotroph described in the present paper compared with the *E. coli* ATCC 23798 could be due to genetic differences. The genotype of ATCC 23798 is not known, but the parent strain is described as having been mutagenized with *N*-methyl-*N*-nitrosoguanidine (17).

**Table 2:** Growth rates and doubling times of the single and double mutants.

Exp. #	Mutant	Met conc. (μM)	Growth rate (OD/h)	Doubling time (min)
1	*metR*	3	0.0091	76.17
		7	0.014	49.51
		10	0.0148	46.83
		13	0.0149	46.52
		15	0.0157	44.15

1	*metRmetD*	3	0.0016	433.21
		7	0.0022	315.07
		10	0.0067	103.45
		13	0.0109	63.59
		15	0.0124	55.90

2	*metR*	3	0.0105	66.01
		5	0.0122	56.82
		7	0.0127	54.58
		9	0.0136	50.97
		11	0.0141	49.16

2	*metRmetD*	9	0.0057	105.02
		11	0.0073	81.55
		13	0.0093	63.59
		15	0.0097	53.73
		17	0.0098	56.82

Cecal prevalence of the *Salmonella* challenge strain was evaluated, and 100% were positive for the challenge strain in the unvaccinated and *metRmetD* vaccinated groups at the end of the trial, whereas 75 and 40% were positive for the challenge strain in the *P_BAD_*-*mviN* and wild-type vaccinated groups as reported previously, respectively (11). Colonization of ceca was also measured by enumeration of challenge strain colonies on selective agar plates containing NA. Both the *metRmetD* and *P_BAD_*-*mviN* vaccine strains significantly reduced (*P* < 0.01) the number of challenge strain *Salmonella* in the cecal contents when compared with the unvaccinated control group (the means ± SD were 4.71 ± 1.41 log CFU/g for *metRmetD*, 2.62 ± 0.8 log CFU/g for *P_BAD_*-*mviN*, and 6.49 ± 0.61 log CFU/g for the unvaccinated group, partially reported in our previous study of the *P_BAD_*-*mviN* vaccine) (11).

**Figure 4:**
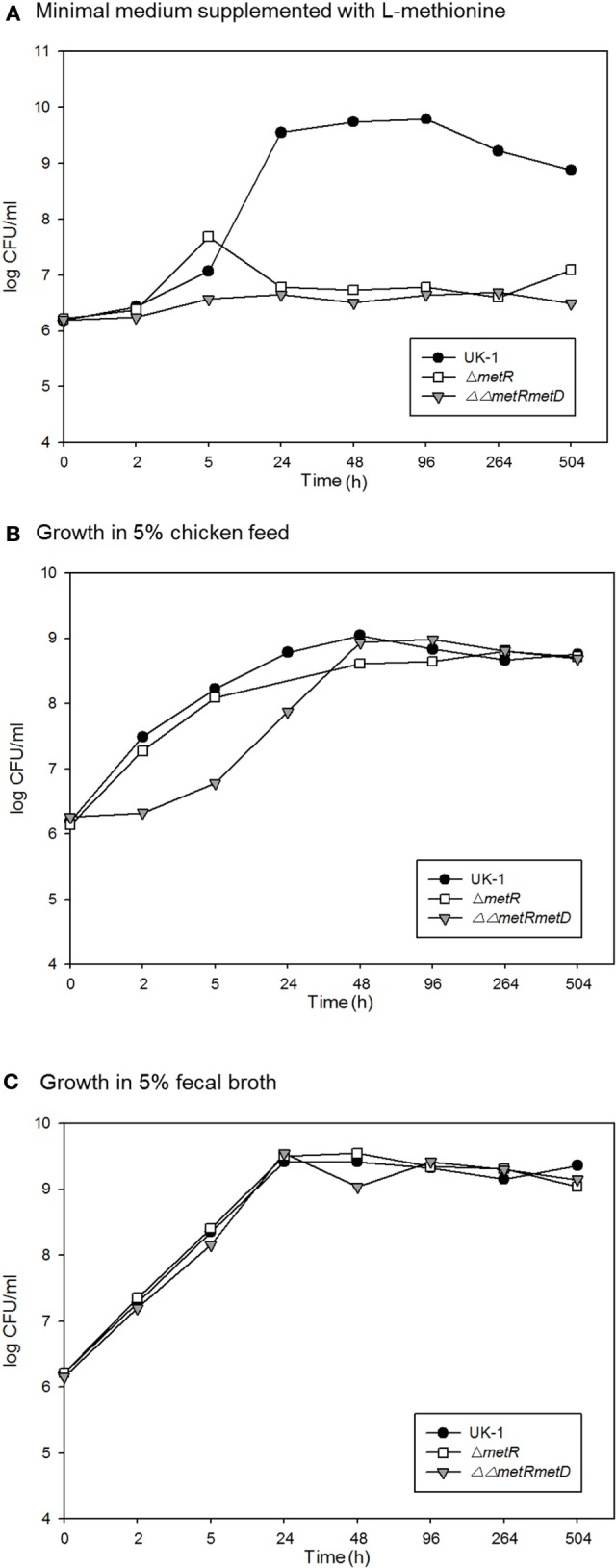
Survival over time of the wild-type parent strain (*S*. Typhimurium UK-1) and methionine auxotrophic mutants in **(A)** M9 minimal medium; **(B)** 5% chicken feed; **(C)** 5% chicken feces.

This suggests the vaccine strains partially protected against challenge strain colonization but based on the greater level of prevalence, the *metRmetD* vaccine candidate strain was not as well cleared by the birds. This is supported by two independent lines of evidence. For one, the survival curves of the methionine mutants in chicken feed and fecal material indicated a high degree of survival in these matrices versus incubation in minimal M9 medium (Figure 4). This may also be reflective of the fact that chickens vaccinated orally with the ΔΔ*metRmetD* mutant exhibited elevated levels of serum IgG binding specifically to *Salmonella* proteins in ELISA relative to an attenuated mutant strain, *P_BAD_*-*mviN* and the unvaccinated group. The *metRmetD* mutant had a mean titer of 7840 ± 1711, while the *P_BAD_*-*mviN* strain had a mean titer of 4520 ± 1544, and the unvaccinated group mean titer was 1700 ± 352.5 (11).

Given the superior immune response of the *metRmetD* mutant, this strain may warrant further research as a vaccine strain. However, this mutant does not appear to be easily cleared out by the inoculated birds and this could be problematic from an environmental contamination standpoint. There are possible remedies for this. To eliminate this problem and further reduce intracellular survival, further investigations may be required that eliminate some of the low-affinity methionine transporters in combination with the knock-out of the high affinity transporter *metD* reported here. Finally, different genes involved in methionine biosynthesis could also be targeted in addition to the transport genes. For example, *S*. Gallinarum *met*C mutants have been shown to possess diminished virulence capabilities, lowered levels in reticuloendothelial organs and competitiveness defects in challenged birds (18).

In conclusion, new vaccine strains (Δ*metR* single mutant and ΔΔ*metRmetD*) were constructed in this study. The methionine auxotroph ΔΔ*metRmetD* generated a greater *Salmonella*-specific serum IgG level and reduced the level of *Salmonella* in cecal contents of approximately 100-fold relative to the unvaccinated control group. Particular combinations of methionine biosynthesis and transport mutants could result in optimal vaccine candidates that can be retained sufficiently to stimulate an optimal immune response but yet easily cleared via dietary manipulation.

## Author Contributions

PR performed experiments, drafted the manuscript, collected test data, and analyzed the data. SK drafted and revised the manuscript. PR, SP and SR designed the study and revised the manuscript.

## Conflict of Interest Statement

The authors declare that the research was conducted in the absence of any commercial or financial relationships that could be construed as a potential conflict of interest.
